# Inequality of opportunities in health and death: an investigation from birth to middle age in Great Britain

**DOI:** 10.1093/ije/dyaa130

**Published:** 2020-10-04

**Authors:** Damien Bricard, Florence Jusot, Alain Trannoy, Sandy Tubeuf

**Affiliations:** 1 IRDES, Paris, France; 2 PSL, Université Paris-Dauphine, LEDA-LEGOS, Paris, France; 3 CNRS, EHESS, Centrale Marseille, AMSE, Aix-Marseille University, Marseille, France; 4 Institute of Health and Society (IRSS) and Institute of Economic and Social Research (IRES), Université catholique de Louvain, Brussels, Belgium

**Keywords:** Childhood, equality of opportunity, health inequality, longitudinal, self-assessed health, mortality

## Abstract

**Objective:**

We assess the existence of unfair inequalities in health and death using the normative framework of inequality of opportunities, from birth to middle age in Great Britain.

**Methods:**

We use data from the 1958 National Child Development Study, which provides a unique opportunity to observe individual health from birth to the age of 54, including the occurrence of mortality. We measure health status combining self-assessed health and mortality. We compare and statistically test the differences between the cumulative distribution functions of health status at each age according to one childhood circumstance beyond people’s control: the father’s occupation.

**Results:**

At all ages, individuals born to a ‘professional’, ‘senior manager or technician’ father report a better health status and have a lower mortality rate than individuals born to ‘skilled’, ‘partly skilled’ or ‘unskilled’ manual workers and individuals without a father at birth. The gap in the probability to report good health between individuals born into high social backgrounds compared with low, increases from 12 percentage points at age 23 to 26 at age 54. Health gaps are even more marked in health states at the bottom of the health distribution when mortality is combined with self-assessed health.

**Conclusions:**

There is increasing inequality of opportunities in health over the lifespan in Great Britain. The tag of social background intensifies as individuals get older. Finally, there is added analytical value to combining mortality with self-assessed health when measuring health inequalities.

**Key Messages**

- Inequality of opportunities in health prevail at all ages in Great Britain and this is true even when using a single indicator of social background—father’s occupation.
- As individuals age, the health gap widens between the health distributions of the most and least advantaged social backgrounds.
- The gap in the probability to report a good health status reaches more than 25 percentage points after age 50 years and differences are more marked when mortality is combined with self-assessed health to measure health status.
- The worst social background tag, in terms of health disadvantages, is that of being born to a family without a father or a father in ‘unskilled’ or ‘partly skilled’ work.

## Introduction

In the past 20 years, numerous empirical studies have evaluated the magnitude of health inequalities between socio-economic groups.^1–4^ Most of this literature usually focuses on health status at only one age or on life expectancy. Life course epidemiology has proposed to explain health inequalities in adulthood by the long-term biological, behavioural and psychosocial processes acting during gestation, childhood, adolescence, early adult life and across generations.^5–7^ In this paper, we focus on the health trajectory from birth to adulthood alone. Since health status is an evolving outcome,^8^^,^^9^ it is important to stretch the snapshots of health inequalities over a lifetime. Few studies have considered health inequalities over a lifetime at different ages or for different age cohorts.^10–12^ They have mainly shown that socio-economic health inequalities increase with age until a certain age from which they decrease because of a population selection effect. Here, we document the worsening effect of inequality of opportunities in Great Britain, one of the most egalitarian countries in terms of nationalized health care.

Measuring health inequalities over a lifetime requires following individuals from birth to death and so, we need to account for the problem of sample selection due to mortality. In general, empirical studies on inequalities separately consider health indicators and do not combine general health measures with mortality indicators. Some studies have used synthetic health indicators over the lifecycle, such as healthy life expectancy, combining health status and mortality.^13^^,^^14^ They consist of population-based health indicators and aggregate several individuals’ health statuses and mortality risk levels at each age within a population or specific group. Such population-based health indicators are therefore inappropriate to measure health inequalities between individuals over the lifecycle. Using individual-based data from a general population survey, Petrie *et al*.^15^ have incorporated death as a health state along with morbidity indicators and showed that it strongly affects the longitudinal analysis of health inequalities.

This paper presents an original study of inequality of opportunities in health, which are the most unjust inequalities. The contribution of the paper is 4-fold. First, the use of a normative approach brings a new perspective on health inequalities considering fairness within the life-course and provides relevant long-term elements to motivate public health policies.^16^ Second, we use cumulative distribution functions (CDFs) to trace the evolution of inequality of opportunities in health over the lifecycle. CDFs have the advantage of being a validated tool for the measurement of inequality of opportunities in many outcomes.^17^ CDFs allow a synthetic and complete description of inequality accounting for the discrete nature of health indicators. They can be compared at different ages using a non-parametric method based on stochastic dominance to show the tag of social background on health overall from birth. Third, we evaluate over the longest lifespan possible using the 1958 National Child Development Study (NCDS), which is the longest birth cohort data available worldwide, providing the health status of a sample of individuals at several ages. Lastly, we consider the complete health trajectory of individuals combining, in a consistent manner, self-assessed health and mortality information to measure health status.

## Conceptual framework

The question of inequality of opportunities has become key to the study of inequalities in health, as well as in other outcomes.^16^^,^^18^^,^^19^ The equality of opportunity theory calls for a normative understanding of health determinants.^20–22^ More generally, childhood characteristics are considered as socially or morally unacceptable sources of inequality.^23–25^ Any difference in the distribution of health in adulthood according to social background is considered an inequality of opportunities in health. The concept of inequality of opportunity distinguishes between legitimate and illegitimate sources of inequality. Legitimate inequalities are due to factors for which the individual can be held responsible, whereas the latter stem from factors beyond the individual’s control. In the terminology of Roemer, these are efforts and circumstances, respectively.^18^^,^^21^ While circumstances are usually proxied by social background, health-related lifestyles have been used to measure efforts in health.^19^ The typical ethical prescription is that inequalities due to circumstances should be compensated for, whereas those due to efforts, and hence ‘legitimate’, should be respected.^16^ We do not elaborate further here about how these principles should be adapted to the health sphere and refer to relevant literature^18^^,^^19^^,^^21^ for additional discussions on that issue. It is however important to underline that most studies in epidemiology that examine the relationships between early childhood circumstances and/or parental characteristics and health could be interpreted through the lens of equality of opportunity theory. According to Bartley^26^ (p. 186) ‘new and important advances in this kind of thinking has linked life-course ideas to ideas from philosophy about individual responsibility versus the force of circumstances’.

Such a conceptual framework influences empirical analyses and often relies upon non-parametric methods. Here, we adopt an *ex ante* perspective for measuring inequalities of opportunities;^27^ we consider father’s occupation at birth as a proxy of the social background and do not use information on health-related lifestyles. This implies that the part of effort that is correlated to father’s occupation is also considered as a circumstance.

Exposure to disadvantaged early life conditions and social background has been associated with poorer health in later life. Four main mechanisms have been discovered in the fields of life course epidemiology as well as social sciences, such as psychology, sociology, demography and economics.^6^^,^^28^^,^^29^ The latency model shows the direct influence of social and family living conditions in childhood on health in adulthood following a latency period.^30–35^ The pathway model relies on social background having an indirect influence on health status in adulthood and subsequent life trajectories, particularly through the transmission of socio-economic status over different generations.^25^^,^^36–44^ According to the risk accumulation hypothesis, poor social and family background combined with social reproduction processes may increase the duration of exposure to disadvantaged conditions. This is associated with long-term health problems and poor social conditions as individuals age.^5–7^^,^^45–47^ Finally, there is evidence of an intergenerational transmission of health-related outcomes such as health disorders,^48–50^ general health^25^^,^^51^^,^^52^ and health-related behaviours.^53–57^

## Methods

### Data sources

We used data from the NCDS, which follows a cohort of 17 500 people born in the same week in March 1958 in Great Britain. We used two alternative samples of data in the empirical analysis: a balanced sample of living individuals, whose data were collected at birth and then collected again at ages 23, 33, 42, 46, 50 and 54 (*n* = 5472), and a sample where individuals have died since 1958 (*n* = 6608) (see Table 1).

**Table 1:** Cohort follow up [source: National Child Development Study—NCDS (1958)]

National Child Development Study (NCDS) 1958	Birth	Wave 4	Wave 5	Wave 6	Wave 7	Wave 8	Wave 9
Collection year	1958	1981	1991	2000	2004	2008	2013
Age, years	Birth	23	33	42	46	50	55
Collected sample	17 415	11 899	10 899	10 830	9057	9279	8670
Dead		883	953	1000	1045	1084	1136
Balanced sample without mortality	5472						
Balanced sample with mortality	6608						

**Table 2:** Distribution of health status and mortality at each wave [source: National Child Development Study—NCDS (1958)]

Self-assessed health	23 years old %	33 years old %	42 years old %	46 years old %	50 years old %	54 years old %
Dead	4.99	6.10	6.88	7.60	8.37	9.24
Poor	0.61	1.24	2.49	5.79	3.88	4.45
Fair	6.29	9.55	11.63	13.82	10.63	11.93
New good					26.49	29.54
Old good	44.27	48.42	49.21	42.81		
Very good					31.88	32.69
Old excellent	43.84	34.68	29.79	29.99		
New excellent					18.74	12.16

### Measures

We considered an ordered and qualitative measure of health status, referred to as self-assessed health, which corresponds in NCDS to individuals' answers to the question ‘how would you describe your health generally?’ Self-assessed health (SAH) is widely used in the literature on health inequalities and data are available for ages 23, 33, 42, 46, 50 and 54 years. A drawback of NCDS is that while the question remained the same across survey waves, the suggested SAH response items changed in the last two waves of the survey. From age 23–46 years, the four health states were (i) poor, (ii) fair, (iii) good, and (iv) excellent, and from age 50 years, a ‘very good’ category was added between ‘good’ and ‘excellent’. Since it is valuable for us to consider individuals over the longest possible lifespan, we considered the SAH variable to have seven potential health states, where individuals reported their health over four possible states in the first four waves and over five possible states in the last two waves. We then collated the old and new states. We renamed ‘good’ and ‘excellent’ in the first four waves as ‘old good’ and ‘old excellent’ and in the last two waves as ‘new good’ and ‘new excellent’. We therefore considered that health statuses could be ranked from the poorest health state to best state as follows: (i) poor, (ii) fair, (iii) new good, (iv) old good, (v) very good, (vi) old excellent and (vii) new excellent.

Measuring health over the lifetime requires considering the health status of an individual from birth until death, therefore we combined the vital status at each time point with SAH (Table 2). In order to account for differences in age at death, we considered death as an additional health status. We assumed that death should be considered as a less desirable health status than ‘very poor’ in self-assessed health, from a normative viewpoint. Consequently, our health indicator scale had eight items from ‘dead’ to ‘new excellent’. Although we could precisely measure the mortality rate from birth to age 23 years in the original sample of the cohort study, there is attrition in the subsample of living individuals included in the balanced sample. We therefore adjusted the mortality rate using a weighting procedure in order to generate an appropriate mortality rate in this subsample. Hence, the mortality rate in the balanced sample (when dead individuals are included) was comparable to the mortality rate of the initial cohort but accounted for attrition in the survey from age 23 years.

Social background was measured by the father’s occupation at the time of birth. The choice of father’s occupation as a single circumstance was motivated by previous research using NCDS and showing that father’s occupation is a leading determinant of health.^58^^,^^59^ Similarly, a recent paper by Jivraj *et al*.^60^ using NCDS included father’s occupation as a single confounding variable to measure childhood social class in order to account for neighbourhood selection across the life course. Father’s occupation is available following the registrar general's class scheme, which indicates employment in six possible fields: professional (I), managerial and technical (II), skilled non-manual (III n. m), skilled manual (III m), partly skilled (IV), and unskilled (V) professions. A seventh category was added if the mother reported no male figure in the household at the time of birth. The distribution of the sample according to social groups is available in Table 3.

**Table 3:** Distribution of father’s professional status [source: National Child Development Study—NCDS (1958)]

Father’s professional status	Freq.	All (%)
I—Professional	324	4.90
II—Managerial/technical	932	14.10
III n. m—Skilled non-manual	665	10.06
III m—Skilled manual	3179	48.11
IV—Partly skilled	721	10.91
V—Unskilled	487	7.37
No father at birth	300	4.54

### Assessing inequality

We tested the presence of inequality of opportunities in health between individuals using a non-parametric approach. The ordered discrete nature of the combined SAH and mortality indicator has the advantage of allowing simple comparisons of health status at each age. The use of a non-parametric approach, based on CDFs and dominance tools, permitted us to account for all the ordered health states and maximize the use of all health and mortality information that is available.

The use of non-parametric methods to assess inequality of opportunities originates from Lefranc *et al*.^17^ and was firstly applied in a health context in Trannoy *et al*.^25^ Evidence of inequality of opportunities relies on the comparison of cumulative distribution functions of the health outcome according to social background — here the father's occupation, which represents ‘circumstances’, according to Roemer.^21^ It is assumed that being born in a particular family is equivalent to getting a lottery ticket whose winnings will only be known later on. The CDF of health status of individuals born to a specific social background (the conditional CDF) gives the probability of not reaching a given health status (for example, death or poor SAH, etc.). In this context, the CDF can be described as the ‘misfortune curve’; the lower, the better. Hence, the conditional CDFs for all backgrounds summarize all the information about the distribution of opportunities in health for people who grew up in different social backgrounds.

We say that there is inequality of opportunities if there are at least two backgrounds for which one CDF is statistically significantly higher than the other. It is much more demanding for a CDF to be higher than it is for the conditional expectation. The price to pay for a robust analysis based on the full probability distribution is that we may not be able to conclude in all cases. For example, if the CDFs cross or the gaps are tiny, it does not allow a judgment. We then need a statistical test to rank the conditional CDFs, a typical situation of first-order stochastic dominance.

Empirically, the inference procedure relies on pairwise tests of equality of distribution for stochastic dominance of CDF based on one-sided Kolmogorov–Smirnov (KS) tests, which are appropriate with discrete variables. The null hypothesis is that one distribution is always above or equal to the other distribution, and if the KS statistic is small or the *P*-value is high then we cannot reject the null hypothesis.

We present the CDFs of SAH with and without mortality according to fathers’ occupation at each age as a graphical demonstration of the dominance. We then complete this graphical intuition with the significance level of the KS tests of the pairwise differences between distributions.

## Results

### Cumulative distribution functions

**Figure 1:**
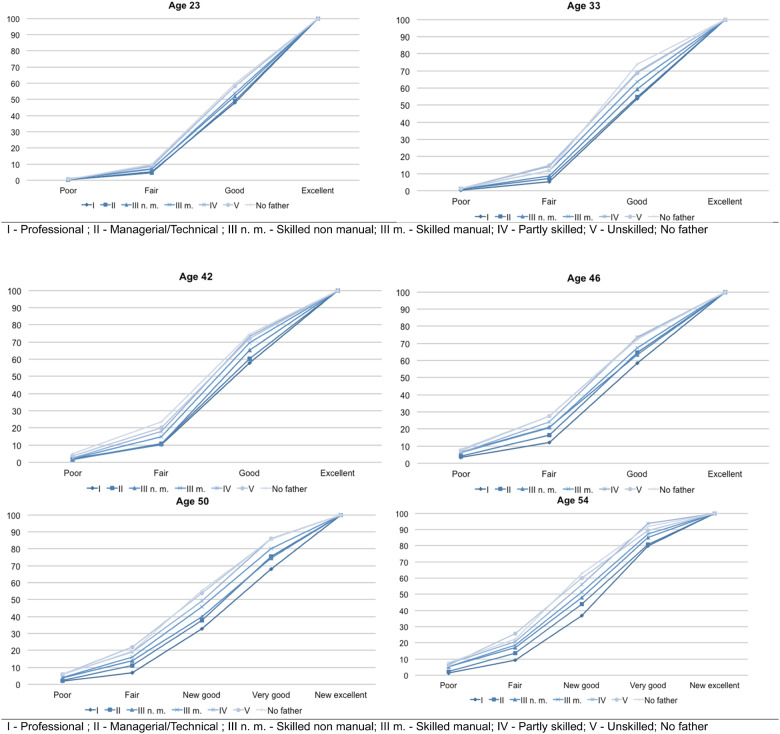
Cumulative distribution functions of self-assessed health according to fathers’ professional status (without mortality) at each age [source: National Child Development Study—NCDS (1958)]. The six graphs represent the cumulated distribution functions of the health status items of the seven possible father's professional status at each age. At age 33 years, the proportion of individuals who report a ‘fair’ health is 14% among sons of ‘partly skilled’ (IV) or ‘unskilled’ (V) manual workers whereas it is only 5% among sons of ‘professionals’ (I). In other words, cumulative distribution functions represent the distribution of the misfortune of health according to father’s professional status

**Figure 2:**
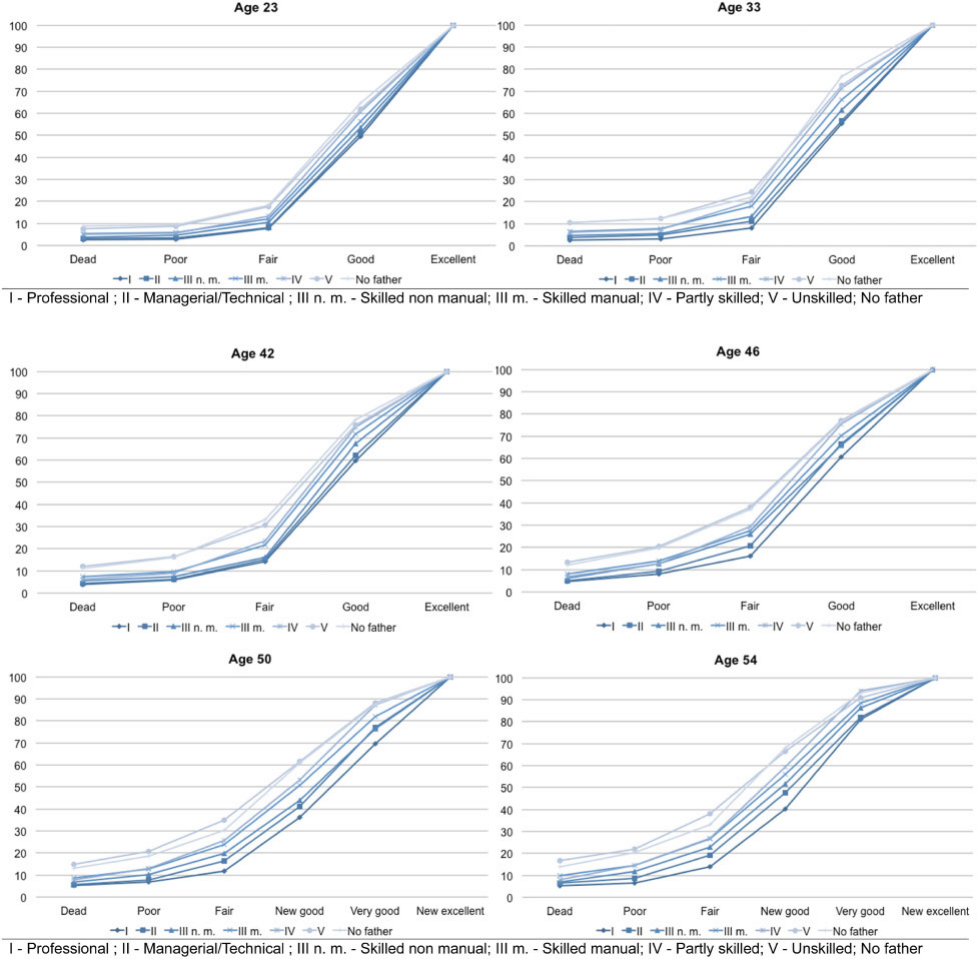
Cumulative distribution functions of self-assessed health according to fathers’ professional status (with mortality) at each age [source: National Child Development Study—NCDS (1958)]. The six graphs represent the cumulated distribution functions of the health status items of the seven possible father's professional status at each age. At age 23 years, the proportion of individuals who have died is <10% regardless of the father's professional status. However it is graphically noticeable that the proportions of premature death at age 23 years among sons of ‘partly skilled’ (IV) or ‘unskilled’ (V) manual workers and individuals without a father at birth are slightly higher than amongst descendants of other father's professional status. Similarly the probability to report a health status as ‘fair’ at age 23 years equals 17.8% for individuals without a father at birth whereas it equals 7.7% for sons of ‘professionals’ (I). In other words, cumulative distribution functions represent the distribution of the misfortune of health according to father’s professional status

Figures 1 and 2 graphically compare the CDFs of health status, respectively, when measured by SAH with and without mortality. Figure 1 shows inequality of opportunities in health according to the father’s occupation at each of the six ages. At age 23 years, the distributions are grouped, whereas they slowly separate over the lifetime, drawing a social gradient in health related to the father’s occupation. At all ages, we observe a gap between the health distribution of individuals who had no father at birth and the distributions of individuals born to a father in the top two occupations, and to a lesser extent, to those born to a non-manual skilled worker. The gap increases between ages 23 and 50 years. For example, the gap in the probability to be in good health between individuals born to a ‘professional’ and those whose father was absent at birth is 26 percentge points (hereafter p.p.) at age 54 years, whereas it was only 12 p.p. at age 23.

When we include death as the worst health state, the CDFs are flatter to the left until age 42 years (Figure 2). This comes from the inclusion of child and adolescence mortality rates and shows that premature mortality is more frequent than reporting a poor health status at younger ages, and this is true across social classes. The gap in reporting good health between individuals born to a ‘professional’ and those without a father at birth is 15 p.p. at age 23 years (12 p.p. when compared with individuals born to an ‘unskilled’ father), which increases to 25 p.p. at age 50 years and 28 p.p. at age 54 years (respectively 25 p.p. at age 50 years, and 26.5 p.p. at age 54 years when compared with individuals born to an ‘unskilled’ father). However, the health distribution of individuals born to a ‘skilled manual’ or ‘partly skilled’ father does not clearly separate from both groups and often crosses other distributions from one age to the other. There is an apparent social gradient in health according to the father’s occupation across all ages. The father’s occupation clearly divides the population into two groups, especially from age 33 to 50 years: individuals born to a father in the top three occupations, who are in better health, and individuals born to an ‘unskilled‘ father or without a father, who are in poorer health; this is apparent at all ages.

### Inference tests

The one-sided KS tests in Tables 4 and 5 confirm the existence of inequalities of opportunity in health according to the father’s occupation. The results show that the distributions of health in adulthood of people born to a ‘professional’ or ‘managerial/technical’ father dominates that of those born to a ‘partly skilled’ or ‘unskilled’ worker or who did not have a father at birth (KS tests: *P* < 0.05 at age 23 years and *P* < 0.01 at ages 33–54 years). When mortality is included as the worst possible health state, the KS tests level of significance increases to *P* < 0.01, regardless of age.

**Table 4:** Lifecycle dominance tests according to fathers’ professional status (without mortality) [source: National Child Development Study—NCDS (1958)]^a^

Column dominates row^b^	I	II	III n. m	III m	IV	V	No father
23, 33, 42, 46, 50, 54 years old							
I							
II	?, ?, ?, ?, ?, ?						
III n. m	?, ?, ?, F, ?, F^*^	?, ?, ?, ?, ?, ?					
III m	?, F^*^, F^*^, F, F^*^, F^*^	?, F^*^, F^*^,?, F^*^, F^*^	? , ?, ?, ?, F,?				
IV	F, F^*^, F^*^, F^*^, F^*^, F^*^	F^*^, F^*^, F^*^, F^*^, F^*^, F^*^	?, F^*^, F, F^*^, F^*^, F	?, F, ?, F, F, F			
V	F, F^*^, F^*^, F^*^, F^*^, F^*^	F, F^*^, F^*^, F^*^, F^*^, F^*^	?, F, F, F, F^*^, F^*^	?, ?, ?, ?, F, F^*^	?, ?, ?, ?, ?, ?		
No father	F, F^*^, F^*^, F^*^, F^*^, F^*^	F, F^*^, F^*^, F, F^*^, F^*^	?, F^*^, F^*^, ?, F^*^, F^*^	?, F,?, ?, F, F^*^	?, ?, ?, ?, ?, ?	?, ?, ?, ?, ?, ?	

a F^*^ represents first order stochastic dominance (FOSD) at 1% (the *P*-value of the one-sided KS test of the difference between the two distributions is <0.01); F represents FOSD at 5% (the *P*-value of the one-sided KS test of the difference between the two distributions is <0.05); ? indicates that we cannot conclude on dominance (the *P*-value of the one-sided KS test of the difference between the two distributions is >0.05).

b The one-sided KS test is read horizontally, the distribution of self-reported health of people born to a father who was in professional work (I) dominates at first order the distribution of self-reported health of people born to a father who had skilled non-manual work (III n. m) at the level of significance *P* < 0.05 at age 46 years at and at the level of significance *P* < 0.01 at age 54 years, however we cannot conclude on dominance at ages 23, 33, 42 and 50 years.

Note: For the sake of clarity we only report the dominance relationships comparing column against row, however we also tested the dominance relationships comparing row against column to infer the direction of the dominance relationship.

**Table 5:** Lifecycle dominance tests according to father’s professional status (with mortality) [source: National Child Development Study—NCDS (1958)]^a^

Column dominates row^b^	I	II	III n. m	III m	IV	V	No father
23, 33, 42, 46, 50, 54 years old							
I							
II	?, ?, ?, ?, ?, ?						
III n. m	?, ?, ?, F, ?, F^*^	?, ?, ?, ?, ?, ?					
III m	?, F^*^, F^*^, F^*^, F^*^, F^*^	F, F^*^, F^*^, F^*^, F^*^, F^*^	?, ?, F, ?, F^*^, ?				
IV	F^*^, F^*^, F^*^, F^*^, F^*^, F^*^	F^*^, F^*^, F^*^, F^*^, F^*^, F^*^	F, F^*^, F, F^*^, F^*^, F	?, F, ?, F, F, F			
V	F^*^, F^*^, F^*^, F^*^, F^*^, F^*^	F^*^, F^*^, F^*^, F^*^, F^*^, F^*^	F, F^*^, F^*^, F^*^, F^*^, F^*^	?, F, F^*^, F^*^, F^*^, F^*^	?, ?, F, F, F^*^, F^*^		
No father	F^*^, F^*^, F^*^, F^*^, F^*^, F^*^	F^*^, F^*^, F^*^, F^*^, F^*^, F^*^	F^*^, F^*^, F^*^, F^*^, F^*^, F^*^	F, F^*^, F^*^, F^*^, F^*^, F^*^	?, ?, F, ?, ?, F	?, ?, ?, ?, ?, ?	

a F^*^ represents first order stochastic dominance (FOSD) at 1% (the *P*-value of the one-sided KS test of the difference between the two distributions is <0.01); F represents FOSD at 5% (the *P*-value of the one-sided KS test of the difference between the two distributions is <0.05); ? indicates that we cannot conclude on dominance (the *P*-value of the one-sided KS test of the difference between the two distributions is >0.05).

b The one-sided KS test is read horizontally, the distribution of health (self-assessed health combined with mortality) of people born to a father who was in professional work (I) dominates at first order the distribution of health (self-assessed health combined with mortality) of people born to a father who had skilled manual work (III m) at the level of significance *P* < 0.01 at age 33, 42, 46, 50 and 54 years, however we cannot conclude on dominance at age 23 years.

Note: for the sake of clarity we only report the dominance relationships comparing column against row, however we also tested the dominance relationships comparing row against column to infer the direction of the dominance relationship.

The results also show that the distributions of health of individuals born to a father in ‘skilled non-manual’ and ‘skilled manual’ work significantly dominate those of individuals born to a ‘partly skilled’ or ‘unskilled’ worker or those who did not have a father at birth only in older age (age 50 and 54 years). When mortality is included, the distributions of health of individuals born to a ‘skilled non-manual’ father are always in better health than individuals of ‘partly skilled’ or ‘unskilled’ worker or who did not have a father at birth (KS tests: *P*≤ 0.05). Furthermore, the number of dominance relationships between the distributions, as well as the level of significance in the differences between those distributions, increase.

## Discussion

This analysis provides evidence of inequality of opportunities in health at all ages, favouring individuals born to a father in ‘professional’, ‘managerial/technical’ and ‘skilled non-manual’ positions in Great Britain. There is a health disadvantage of having no father at birth or a father who is ‘unskilled’ or ‘partly skilled’ over the lifetime. Inequality of opportunities in health is found to increase with age and the diagnosis worsens when premature death is taken into account. This outcome prevails despite vigorous action taken in Britain to fight health inequalities.^61–63^

Our study results align with the literature in life-course epidemiology, showing that individuals from a less well-off social background report poorer health at all ages and are more likely to die prematurely in Great Britain.^5^^,^^6^ The principal novelty of the paper is to provide a simple quantification of the increasing unfair health inequalities over a lifetime, combining self-assessed health and mortality consistently. This finding is consistent with the work of van Kippersluis *et al*.^64^ which suggests an increase in income-related health inequalities until retirement age in the UK, however it does not confirm the predictions of the theoretical modelling proposed by Galama and van Kippersluis.^65^ Another novelty is the use of a robust non-parametric method allowing us to mobilize all the response items of the SAH instead of summarising them in a binary indicator. We do not throw any piece of information away thanks to the chosen statistical methodology. In that sense, our statistical approach can be described as comprehensive. Additionally, we offer an original and simple way to combine an ordered discrete health indicator with mortality. The additional advantage of including mortality as a health indicator is that it allows us to work with a larger sample and accounts for the selection bias of premature mortality related to social status.

Our results are particularly striking since we identify substantial differences in health status until late in adulthood, using only one indicator of childhood circumstances. This is a minimalistic identification of inequalities of opportunities in health but it is robust. Although one might like to see further circumstances being considered, a difficulty of the dominance analysis is that it assumes the availability of large samples to perform inference tests. If we intersect several circumstances, then sample size substantially reduces, and the dominance statistical inference tests cannot be useful any longer. Since equality of conditional CDFs is a necessary condition for equality of opportunity, even if circumstances are not fully described we can say that equality of opportunity in health is violated when the KS test shows significant differences between CDFs.^17^ This will remain true if we had the possibility to measure circumstances perfectly.

Another limitation comes from the 1958 NCDS having a singular structure with the different waves not being equidistant in time. While there is a 4-year interval between the two last sweeps, there were about 10 years between the previous waves. It was not possible in our non-parametric approach to account for this effect.

Inequality of opportunities in health and death deepens with age, at least up to mid-life. Our study does not provide new information about the mechanisms behind this phenomenon, but clearly it is an issue that should be further investigated to develop health policy recommendations for reducing health inequalities.

## Funding

We gratefully acknowledge the financial support of the Health Chair, a joint initiative by PSL, Université Paris-Dauphine, ENSAE and MGEN under the aegis of the Fondation du Risque (FDR) to present the work in various conferences. This paper was presented and discussed in a number of workshops and seminars; we would like to thank Jérôme Wittwer, Brigitte Dormont, Erik Schokkaert, Aki Tsuchiya, Rhiannon Tudor-Edwards and John Mullahy for their comments and suggestions. We thank four anonymous reviewers for their valuable comments and suggestions.

## Conflict of interest

None declared.
